# Comparing the technical quality and clinical outcomes of root canal treatment on immature permanent incisors in children: a retrospective evaluation of three bioceramic plug materials

**DOI:** 10.1007/s40368-024-00941-3

**Published:** 2024-09-10

**Authors:** Christopher C. Donnell, Pathanjali Kandiah

**Affiliations:** 1 Department of Paediatric Dentistry, Charles Clifford Dental Hospital, Sheffield Teaching Hospitals NHS Foundation Trust, 76 Wellesley Road, Sheffield, S10 2SZ UK; 2 Academic Unit of Oral Health, Dentistry and Society, School of Clinical Dentistry, University of Sheffield, Sheffield, UK

**Keywords:** Apexification, Immature incisors, Paediatric dentistry, Technical quality, Clinical outcome, Bioceramics

## Abstract

**Purpose:**

To assess the technical quality and clinical outcomes of non-surgical endodontic treatment of immature permanent incisor teeth with three different bioceramic plug materials and highlight variables which may influence treatment and quality outcomes.

**Methods:**

This cross-sectional analysis forms part of a retrospective service evaluation of the technical quality and clinical outcome of orthograde root canal treatment carried out in the Paediatric Dentistry Department of Charles Clifford Dental Hospital (United Kingdom). Twenty-five cases were identified chronologically, using the electronic patient record system, for three bioceramic plug materials: Mineral Trioxide Aggregate (MTA), Biodentine, and TotalFill Putty. All radiographs were analysed using standard conditions. Intra- and inter-examiner agreement was calculated using Kappa and weighted Kappa tests. Data were collected using a data collection tool, entered into Microsoft Excel, and analysed using descriptive statistics, exploratory analysis with Chi-squared tests, and multivariable analyses (logistic regression).

**Results:**

At 12-month review, the success rate for each apexification material was MTA (84%), Biodentine (88%), and TotalFill Putty (92%). MTA had the highest frequency of post-operative coronal discolouration, with Biodentine most associated with apical extrusion. A number of variables and trends that affect the clinical outcome were identified, including the presence of pre-operative resorption, the number of operators involved in treatment, the number of appointments to complete treatment, as well as how non-use of local anaesthetic during apical plug placement had no adverse effect on technical quality or clinical outcome.

**Conclusions:**

MTA, Biodentine, and TotalFill Putty are highly effective apexification materials which produce excellent clinical outcomes. As such, logistical and situational factors, such as continuity of care from operators with increased levels of experience, skill and ability, rather than material choice, may be more prognostic regarding the technical quality and clinical outcome of immature endodontic treatment. Further high-quality evidence is required.

**Supplementary Information:**

The online version contains supplementary material available at 10.1007/s40368-024-00941-3.

## Introduction

Traumatic dental injuries (TDIs) affect more than 1 billion people worldwide and are currently the fifth most frequent disease or injury identified in patients (Abbott 2018). In children, TDIs represent the most frequent cause of pulpal non-vitality in immature permanent incisors in children aged 8–12 years (Duggal et al. 2017, Levin et al. 2020). It is estimated that one-in-five children will sustain trauma to their developing permanent incisors, with around 6% of all TDIs becoming non-vital and requiring endodontic treatment (Carrotte 2005).

If TDIs damage the Hertwig’s epithelial root sheath (HERS) of immature teeth, subsequent pulpal necrosis may arise and root development may be arrested (Moore et al. 2011). Immature teeth are newly erupted teeth with open apices which take around three more years to complete root development (Singh et al. 2017). Root development is through the continuous deposition of dentine and cementum by stimulation and differentiation of HERS and surrounding progenitor cells, and hence, any interference can produce a compromised crown–root ratio, thin root walls, and a wide-open apex lacking an apical stop (Andreasen et al. 2011, Duggal et al. 2017). Treating these teeth can be time-consuming and technically complex due to challenging debridement as a result of the inverse canal taper, alongside problematic determination of working length due to lack of an apical stop (Duggal et al. 2017, Kim and Chandler 2013). An apical barrier is needed to allow placement and containment of the root filling—which requires an average of two visits over a period of 1–2 months (Mackie 2010).

Treatment can be further complicated by higher levels of dental fear and anxiety in children, typically around aspects of local anaesthesia (LA), which can create issues with patient cooperation and create distress for both patients and parents (Chen et al. 2020, Noble et al. 2020). As such, challenges with patient cooperation are important to document as they may also influence the quality of the endodontic treatment (Clarke et al. 2015), in addition to children being at risk of dental burnout during prolonged courses of treatment associated with TDIs (Donnell 2023)—children with a TDI often present with a caries free dentition, and hence, every stage of endodontic treatment is potential alien territory for the uninitiated patient (Phillips et al. 2020).

Following seminal work by Parirokh and Torabinejad (2010), there has been a paradigm shift in treatment protocols, as bioceramics have now replaced calcium hydroxide as the material of choice for apexification procedures of immature permanent teeth, with MTA the gold standard against which new materials must be tested (Dania and Prisinda, 2022). Traditionally, apexification has been achieved through placement of non-setting calcium hydroxide (Ca(OH)_2_) paste; however, this technique involves repeated and protracted dressing of the root canal, anywhere from 5 to 20 months (12.9 months on average) in duration (Andreasen et al. 2011, Campanella et al. 2020). The unpredictable and prolonged timeframe of calcium hydroxide apexification risks loss of patient cooperation, as well as being linked with an increased risk of bacterial contamination, through the microleakage of temporary restorations, and root fracture (Andreasen et al. 2002, Dominguez Reyes et al. 2005). A number of new materials have emerged onto the commercial market in recent years, demonstrating the rapid advance of material science in dentistry, with a recent ex-vivo study confirming the suitability of calcium silicate cements (CSCs) for root-end closure—mineral trioxide aggregate (MTA), Biodentine, and TotalFill Putty (termed EndoSequence Putty in North America [as both materials have the same chemical composition]) (Tran et al. 2016) (Table 1).

**Table 1:** Comparison of apexification materials

	Calcium hydroxide	ProRoot MTA	Biodentine	TotalFill Putty
Estimated Cost Per Tooth*	£22	£39	£30	£26
Appointments	Multiple	Single Visit	Single Visit	Single Visit
Setting Time	N/A	3–4 Hours	9–12 Minutes	20 Minutes
Mixing	Syringe	Manual / Capsule	Manual / Capsule	Syringe
Cement Type	N/A	Portland Cement	Tricalcium Silicate	Tricalcium Silicate
Placement	Syringe	Carrier / Paper Points	Plugger	Plugger
Radiopacifier	Barium Sulphate	Bismuth Oxide	Zirconium Oxide	Tantalum Oxide
Discolouration	Low Risk	Increased Risk	Low Risk	Low Risk
Handling	Easy	Difficult	Moderate	Moderate/Easy
Radiopacity Visualisation	Poor – Below ISO Standards	Good – Above ISO Standards	Poor – Below ISO Standards	Good – Above ISO Standards

*N/A*: Not Applicable, *ISO*: International Standards Organisation, *MTA*: mineral trioxide aggregate, *(Ca(OH)*_*2*_*)*: Calcium Hydroxide, *£*: British Pound Sterling (GBP)

^*^The estimated cost-per-tooth has been calculated using: volumetric root canal space measurements presented in the literature of maxillary central incisor teeth (Wang et al. 2023); a hypothetical minimum apical plug length of 4mm as recommended in the literature (Torabinejad et al. 2018); the volume of calcium hydroxide (Ca(OH)_2_) used per visit to fill the canal space (Shetty et al. 2021, Thomas et al. 2023); the average number of visits previously cited for Ca(OH)_2_ apexification (Campanella et al. 2020); and the price and product details of the materials listed online (https://www.henryschein.co.uk/gb-en/dental-gb/c/endodontics)

Several studies have demonstrated the benefits of these CSCs over traditional calcium hydroxide, such as: enhanced compressive strength; low solubility when set; improved sealing ability preventing bacterial ingress; antibacterial to facultative anaerobes; excellent biomineralisation capability, and biointeractivity; and all remain unaffected by contact with tissue fluids and/or blood (Dominguez Reyes et al. 2005, Gartshore 2017, Kaur et al. 2017, Pereira et al. 2021, Shokouhinejad et al. 2012, Torabinejad et al. 2017, Torabinejad et al. 2018). Of course, they also come with some limitations, such as high cost, difficult mixing, and handling characteristics, can cause tooth discolouration, prolonged setting time, and considerable wastage associated with those available in capsular form (Antunes et al. 2016, Ayub and Darcey 2023, Nair et al. 2011, Pires et al. 2021, Torabinejad et al. 2017).

In the United Kingdom (UK), the majority of paediatric dentistry departments currently use TotalFill Putty for apexification procedures, despite no published evidence supporting its use over other materials, with other departments favouring MTA, and a minority using Biodentine. While most settings only use one material, a number have more than one material available, with personal preference the deciding factor when choosing a material, influenced by the previous experience and exposure to various plug materials (Donnell and Kandiah 2022). The cost of TDI management for paediatric patients is an estimated £856 (Wong and Kolokotsa 2004), and hence, knowledge of the clinical and economic benefit of apexification materials will be beneficial for healthcare services, as it will provide information surrounding the most clinically and cost-effective option for use, in general, hospital and salaried dental services (Hadjiantonis 2016).

Although a small number of studies have been carried out in children to support treatment with MTA (Clarke et al. 2015, Gartshore 2017), the majority of studies focus on adult populations, and hence, there is a gap in the literature as little information is available to support and validate the use of Biodentine or TotalFill Putty in the paediatric population (Anjum et al. 2024). There are currently no published studies involving paediatric patients comparing all three materials at any level on the hierarchy of evidence from case reports to randomised control trials (RCTs) (Ravindran et al. 2022, Wikström et al. 2021). This study aims to compare the technical quality and clinical outcomes of non-surgical endodontic treatment of immature permanent incisor teeth with MTA, Biodentine, and TotalFill Putty, and to analyse the effect of different factors involved in treatment which may influence clinical outcomes and technical quality.

## Materials and methods

### Study design

This cross-sectional analysis formed part of a retrospective service evaluation of the technical quality and clinical outcomes of orthograde root canal treatment carried out in the Paediatric Dentistry Clinic of Charles Clifford Dental Hospital (United Kingdom). Any orthograde apexification procedure carried out on an immature permanent incisor tooth in a patient aged 16 years or younger was included for evaluation. Root canal treatment carried out under general anaesthetic was excluded from the analysis, as were all teeth that had a closed apex and did therefore not require apical plug placement. Endodontic treatment was performed by staff members with varying degrees of experience—postgraduate students (PG), specialty registrars (StR), specialty dentists (SD), and consultants (Cons) in paediatric dentistry; dental core trainees (DCTs) perform access and extirpation only, and do not place apical plugs.

The evaluation complied with ethical principles and was registered with the Clinical Effectiveness Unit of the Sheffield Teaching Hospitals NHS Foundation Trust (Ref. 10680). Cases were identified from the electronic patient record system (Lorenzo) using clinical outcome coding data and cross-referenced against the patient’s records including their case history and clinical information to confirm eligibility against the inclusion and exclusion criteria. The first 25 cases were then identified chronologically, for each of the three bioceramic plug materials—Mineral Trioxide Aggregate (MTA) (White ProRoot MTA, Dentsply, Tulsa, OK, USA), Biodentine (Septodont, Saint-Maur-des-Fossés, France), and TotalFill BC RRM Fast Set Putty (FKG Dentaire, Neuchatel, Switzerland). Where a patient had multiple endodontically treated teeth, each tooth was entered as a separate subject.

As this was retrospective data collection of everyday clinical practice, and not that following a prospective RCT, treatment was not performed according to predetermined protocols at each visit. All staff performing treatment, however, have had similar training in instrumentation techniques and perform treatment in line with departmental protocols. While some elements vary, the main procedural steps remain standardised across clinicians—these are outlined in Box 1.

Data were collected using a data collection tool under the headings in Box 2 and entered into Microsoft Excel (Microsoft Excel for Mac Version 16.54 [21101001]) as discrete and continuous nominal data. The categories of each variable were coded to allow for ease of statistical analysis. The data collection tool was piloted on ten patients with slight changes to the layout and order as a result.

Box 1 Standardised departmental apexification procedure

**Table:** 

Initial visit	Obturation visit
- Local anaesthetic (LA) administration via labial infiltration (+/− palatal/lingual infiltration) - Isolation with rubber dam and Oraseal - Conventional access cavity preparation - Necrotic pulp extirpation with appropriate manual instrumentation - Irrigation with 5.25% sodium hypochlorite solution - Manual working length determination (without electronic apex location [EAL]) - Working length periapical radiograph (PAR) - Apical gauging with hand file instrumentation - Further irrigation with 5.25% sodium hypochlorite solution - Canal dried using absorbent paper points - Placement of non-setting calcium hydroxide using a backfill approach from the working length to cemento-enamel junction (CEJ) - Access cavity temporarily sealed using an endodontic sponge, followed by a pink glass-ionomer cement (GIC) restoration (Fuji Triage, GC Europe, Leuven, Belgium).	- Optional LA administration - Isolation with rubber dam and Oraseal - Removal of the temporary dressing and endodontic sponge - Irrigation with 5.25% sodium hypochlorite solution - Re-establish and confirmation of working length without EAL - Further irrigation with 5.25% sodium hypochlorite solution - Canal dried using absorbent paper points - Place first 2–3 increments of plug material at working length and condense with an appropriately-sized apical plugger - Radiographic examination with PAR - If in correct position, placement of further plug material to ensure plug is at least 4mm in length - Backfill obturation of the remaining root canal space using a resin-based sealer and thermoplastic gutta-percha (GP) - Radiographic examination with PAR - Removal of excess GP to level of CEJ - Restoration of tooth with bonded coronal resin (composite) restoration

Box 2 Clinical and radiographic variables measured

**Table:** 

*Clinical variables* Tooth type Age Sex Trauma type Pre-operative pulp condition Primary treatment or re-treatment case Use of local anaesthetic (LA) Operator grade and number of operators Number of appointments Time (days): referral to access (extirpation), access to obturation Cooperation Intra-canal medicament Obturation technique Type of sealer used	*Radiographic variables* Periapical radiolucency (PARL) - Presence (and size [mm]) - Absence - Size of apical area at follow-up o Unchanged o Increasing o Decreasing o Not present (healed) Radiograph grade Apical stop length Distance from radiographic apex Canal space visible beyond apical plug (mm) Canal space between plug and obturation Presence/absence of voids Homogeneity of obturation

### Sample size

A recent systematic review found that the majority of the literature concerning apexification consists of case reports and case series, with very few RCTs, prospective or retrospective cohort studies (Torabinejad et al. 2017). As the chief aim of this study was to determine the proportion of a population demonstrating a characteristic (i.e., satisfactory clinical outcome following apexification), a sample size calculation for a single proportion was performed. In line with the studies of paediatric populations by Gartshore (2017) and Clarke et al. (2015), an estimated proportion for the number of satisfactory endodontic procedures was calculated and set at 80%, based on the previous success rates of non-surgical endodontic treatment in the adult literature (Farzaneh et al. 2004, Friedman et al. 2003, Marquis et al. 2006, Ng et al. 2011a, Ng et al. 2011b). The minimum sample size required for a ±10% margin of error at 95% confidence was 62 patients—this was rounded up to 75 (25 per material), for ease of numerical analysis.

### Radiographic evaluation and outcome assessment measures

All images evaluated were periapical radiographs predominantly taken using the paralleling technique and obtained using Enterprise Imaging (EI) PACS 8.1 software (AGFA Healthcare, Leeds, England). Radiographs were assessed for technical quality and outcome by one examiner who was independent from treatment. To account for the subjectivity of radiographic assessment, a second examiner graded the technical quality and outcome of all radiographs assessed. Examiners were calibrated with 15 scans (20% of the sample size), which were not included in the main study. Evaluation of each image was repeated 2 weeks later, and intra- and inter-observer reliability scores calculated. In line with the previous studies (Ballikaya et al. 2022), the baseline, apical plug, post-obturation, and 12-month follow-up images were viewed on a 24-inch LCD monitor with 1920 × 1080 resolution (Dell, Round Rock, TX, USA), in a dimly lit, quiet room (Harvey and Patel 2020). No time restriction was set, and examiners were able to use a zoom tool and brightness/contrast tool to adjust according to their preferences. In cases where measurements were ambiguous (e.g., distance from plug terminus to radiographic apex of tooth with oblique angled apex), the software measurement tool on the radiographic software was used and calibrated using a previous working length radiograph, where possible. In cases of disagreement the image would be reviewed by a third examiner to obtain a consensus; however, this was not required. Clinical and radiographic outcome assessment measures are described in Box 3 —based on variables identified in previous landmark studies (Ng et al. 2008) and standards set by the European Society of Endodontology (ESE) (Endodontology 2006), British Society of Paediatric Dentistry (BSPD) (Mackie 2010), British Endodontic Society (BES) (Tomson 2022), and European Academy of Paediatric Dentistry (EAPD) (Duggal et al. 2017).

**Box 3** Clinical and radiographic outcome assessment measures

**Table:** 

**Satisfactory radiograph**: at least 2–3 mm of the PA region clearly identifiable* **Satisfactory apical plug length**: at least (but ideally) 4–5mm in length* **Satisfactory distance of apical plug from the radiographic apex** • No space between canal material and canal wall should be seen* • There should be no more than 1 mm length of canal space visible beyond the endpoint of the apical plug* • The prepared root canal should be filled completely unless space is needed for a post* • The prepared and filled canal should contain the original canal* **Homogeneity of root canal filling** • Adequate (homogeneous density, no voids)* • Acceptable (apical seal present but non-homogenous density or voids elsewhere in the canal) Clinical/radiographic outcome at discharge • Healed (Success) - no clinical signs or symptoms and absence of radiolucency • Healing (Success) - no clinical signs or symptoms and radiolucency decreasing in size • Continuing disease state (Survival) - no clinical signs or symptoms but persisting radiolucency (Increased or no decreased in size) • Failure - Symptomatic and persisting radiolucency	

*denotes a satisfactory endodontic treatment

### Statistical analysis

The primary outcome measures for the study were the technical quality and clinical outcomes of immature apexification procedure, expressed in frequency and percentage. Statistical analyses were performed using Statistical Package for the Social Sciences ([SPSS] Mac Version 26.0.0.0; SPSS Inc. Chicago, IL, USA) using descriptive statistics, exploratory bivariate analysis with chi-squared tests (*p*<0.05), and multiple logistic regression analysis to test for associations between selected variables, with a Bonferroni correction used to adjust for the effect of other variables (*p*<0.007 [7 variables]). Intra- and inter-examiner agreement was calculated using Kappa and weighted Kappa tests. Substantial agreement was set at 0.61–0.80, and almost perfect agreement at 0.81–1.00.

## Results

A total of 75 incisor teeth (75 patients) were analysed for the technical quality and clinical outcome of apexification and obturation procedures. No patient evaluated in the study had more than one tooth endodontically treated. The intra- and inter-examiner Kappa values calculated indicated almost perfect agreement; 0.81 and 0.82, respectively. Loss of vitality for all teeth (100%; *n*=75) followed a diagnosis of dental trauma, with a relatively equal distribution of age and sex across all treatment groups (Table 2).

**Table 2:** Basic demographic information for each treatment group

Variable	Category	MTA (N/%)	Biodentine (N/%)	TotalFill Putty (N/%)
Age		Mean – 10.2 (7−12; SD 1.43)	Mean − 10.8 (8−12; SD 1.67)	Mean – 10.6 (8−11; SD 1.91)
Sex	Male	15 (60)	17(68)	13 (52)
	Female	10 (40)	8 (32)	12 (48)
Tooth Type	Maxillary Central Incisor	20 (80)	21 (84)	19 (76)
	Maxillary Lateral Incisor	5 (20)	3 (12)	4 (16)
	Mandibular Central Incisor	0 (0)	1 (4)	2 (8)
	Mandibular Lateral Incisor	0 (0)	0 (0)	1 (4)
Nature of Trauma	Crown Fracture	13 (42)	17 (68)	11 (44)
	Avulsion	5 (20)	4 (16)	5 (20)
	Lateral Luxation	4 (16)	2 (8)	6 (24)
	Subluxation	0 (0)	0 (0)	1 (4)
	Intrusion	0 (0)	0 (0)	1 (4)
	Extrusion	3 (12)	2 (8)	1 (4)
Tooth Status at Presentation	Discolouration	6 (24)	2 (8)	3 (12)
	Mobility	8 (32)	6 (24)	10 (40)
	Sinus	1 (4)	2 (8)	2 (8)
	Swelling	12 (48)	9 (36)	3 (12)
	TTP	11 (44)	15 (60)	16 (64)
	Not Recorded	4 (16)	5 (20)	0 (0)
Overall Cooperation	Good	16 (64)	18 (72)	19 (76)
	Limited	5 (20)	4 (16)	4 (16)
	Not Recorded	4 (16)	3 (12)	2 (8)
Type of Treatment	Primary	24 (96)	24 (96)	25 (100)
	Re-Treatment	1 (4)	1 (4)	0 (0)

*MTA* mineral trioxide aggregate, *TTP* Tenderness To Percussion/Palpation, *SD* Standard Deviation

### MTA

**Fig. 1:**
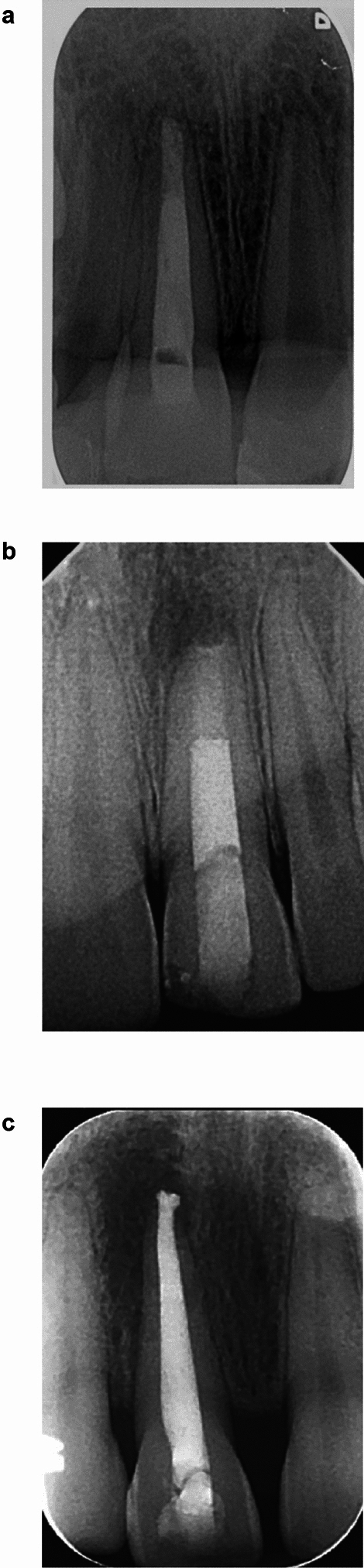
**a** Periapical radiograph demonstrating the desired technical quality of an MTA apical plug and obturation on a maxillary right central incisor (UR1). **b** Periapical radiograph demonstrating the desired technical quality of a Biodentine apical plug and obturation on a maxillary left central incisor (UL1). **c** Periapical radiograph demonstrating the desired technical quality of a TotalFill Putty apical plug and obturation on a maxillary right central incisor (UR1)

Of the 25 cases analysed, the mean age of patients was 10.2 years (7−12; SD 1.43), 15 were male and 10 female (Table 2). Overall average treatment time (new patient appointment to obturation) was 59 days. Of the teeth treated, 20 (80%) were maxillary central incisors and 5 (20%) were maxillary lateral incisors. The most common trauma sustained was complicated crown fracture (*n*=13; 52%), with 96% (*n*=24) primary root canal treatments. Twenty-three patients (92%) had local anaesthetic (LA) for the access visit, compared to only three (12%) having LA for the apical plug and obturation (Table 3). On average, the number of appointments attended was 2.35 and each patient was treated by at least two different clinicians (Table 4). Twenty teeth (80%; 95% CI 67–86%) were classified as meeting the satisfactory quality criteria (Box 3) (Fig. 1a). Of the five cases (20%) deemed unsatisfactory, four (80%) were >1mm from the radiographic apex and all were of non-homogenous density with voids present at the apex. Five apical plugs (20%) were less than 4mm in length. Twelve teeth (48%) had obturation material extending above the level of the cemento-enamel junction (CEJ) into the coronal tooth space. Overall success rate (including survival [unchanged radiolucency]) at 12-month review was 84%. Coronal discolouration was present in seven cases (28%). Four cases (16%) exhibited clinical signs and symptoms of failure, including tenderness to percussion and the presence of swelling and a sinus (Table 4). The presence of clinical signs and symptoms of failure was statistically significant (*p*=0.004) in those cases with increasing periapical radiolucencies (PARL) (*n*=4; 16%). Full details surrounding clinical outcomes are available in ‘Supplementary Table 1’.

**Table 3:** Procedural variables for each treatment group

Variable	Category	MTA (N/%)	Biodentine (N/%)	TotalFill Putty (N/%)
**Intial visit - Access**				
Operator Grade	PG	3 (12)	2 (8)	2 (8)
	DCT	3 (12)	4 (16)	3 (12)
	SD	3 (12)	3 (12)	1 (4)
	StR	7 (28)	9 (36)	11 (44)
	Cons	9 (36)	7 (28)	8 (32)
LA	Yes	23 (92)	24 (96)	25 (100)
	No	1 (4)	0 (0)	0 (0)
	Not Recorded	1 (4)	1 (4)	0 (0)
Irrigant Used	NaOCl	23 (92)	24 (96)	24 (96)
	CHX	1 (4)	0 (0)	0 (0)
	Other	1 (4) - LA	0 (0)	0 (0)
	Not Recorded	0 (0)	1 (4)	1 (4)
Inter-visit Dressing	Ca(OH)_2_	20 (80)	20 (80)	25 (100)
	Ledermix	1 (4)	0 (0)	0 (0)
	Other	0 (0)	0 (0)	0 (0)
	Not Recorded	4 (16)	5 (20)	0 (0)
**Apical plug and obturation visit**				
Operator Grade	PG	1 (4)	2 (8)	0 (0)
	DCT	0 (0)	0 (0)	0 (0)
	SD	3 (12)	4 (16)	0 (0)
	StR	13 (42)	14 (56)	13 (52)
	Cons	8 (32)	5 (20)	12 (48)
LA	Yes	3 (12)	2 (8)	6 (24)
	No	22 (88)	20 (80)	19 (76)
	Not Recorded	0 (0)	3 (12)	0 (0)
Irrigant Used	NaOCl	23 (92)	22 (88)	25 (100)
	CHX	0 (0)	0 (0)	0 (0)
	Other	0 (0)	0 (0)	0 (0)
	Not Recorded	2 (8)	3 (12)	0 (0)
Sealer Used	ZnOE	22 (88)	11 (44)	8 (32)
	AH Plus	0 (0)	11 (44)	13 (52)
	TotalFill BC Sealer	0 (0)	0 (0)	4 (16)
	Not Recorded	3 (12)	3 (12)	0 (0)
Definitive Restoration (Composite)	Palatal Access	8 (32)	12 (48)	12 (48)
	Crown Former	10 (40)	8 (32)	13 (52)
	Free Hand	5 (20)	3 (12)	0 (0)
	Other	2 (8)	2 (8)	0 (0)

*MTA* mineral trioxide aggregate, *PG* postgraduate student, *DCT* dental core trainee, *SD* specialty dentist, *StR* specialty registrar, *Cons* consultant, *Ca(OH)*_*2*_ Calcium Hydroxide, *LA* local anaesthetic, *NaOCl* sodium hypochlorite, *CHX* chlorhexidine, *ZnOE* zinc oxide eugenol, *AH Plus* AH plus® bioceramic sealer, *CEJ* cemento-enamel junction

**Table 4:** Technical quality for each treatment group

Variable	Category	MTA (N/%)	Biodentine (N/%)	TotalFill Putty (N/%)
Number of Operators		Mean 1.4 (1−6; SD 1.03)	Mean 1.5 (1−5; SD 1.29)	Mean 1.7 (1−4; SD 1.48)
Number of Appointments		Mean 2.35 (1−7; SD 1.22)	Mean 2.65 (1−6; SD 1.17)	Mean 2.84 (1−4; SD 1.33)
Key Dates	NPT to Access	Mean Days – 13.2 (0−31; SD 6.23)	Mean Days – 17.5 (0−34; SD 7.68)	Mean Days – 24.2 (0−33; SD 5.29)
	Access to Obturation	Mean Days – 45.7 (0−291; SD 28.3)	Mean Days – 49.2 (0−295; SD 29.1)	Mean Days – 63.2 (0−312; SD 38.1)
	Overall	Mean Days – 58.9 (SD 31.6)	Mean Days – 66.7 (SD 32.6)	Mean Days – 87.4 (SD 37.2)
Plug distance from Radiographic Apex	Optimal	16 (64)	15 (60)	20 (80)
	Short	4 (16)	3 (12)	0 (0)
	Flush	4 (16)	4 (16)	4 (16)
	Over-Extended	1 (4)	3 (12)	1 (4)
Apical Plug Length	Adequate	18 (72)	13 (52)	18 (72)
	Insufficient	5 (20)	3 (12)	0 (0)
	Excess	2 (8)	9 (36)	7 (28)
Obturation	GP above CEJ	12 (48)	10 (40)	6 (24)
Homogeneity	Adequate	18 (72)	16 (64)	20 (80)
	Acceptable	2 (8)	5 (20)	4 (19)
	Inadequate	5 (20)	4 (16)	1 (4)
Discharge Outcome	Healed	15 (60)	16 (64)	18 (72)
	Healing	5 (20)	5 (20)	4 (16)
	Continuing Disease (Survival)	1 (4)	1 (4)	1 (4)
	Failure	4 (16)	3 (12)	2 (8)

*MTA* mineral trioxide aggregate, *NPT* new patient appointment, *GP* gutta-percha, *CEJ* cemento-enamel junction, *SD* Standard Deviation

### Biodentine

Of the 25 cases analysed, mean age was 10.8 years (8−12; SD 1.67), with 17 male and 8 female patients. The mean treatment time from assessment to obturation was over 2 months; 67 days (SD 32.6), with the longest treatment time nearly 10 months (295 days) (Table 2). Maxillary central incisor teeth were the most common tooth treated (*n*=21; 84%), with complicated crown fracture the most common injury sustained (*n*=18; 72%). Only one case (4%) was a re-treatment procedure. The mean number of appointments was 2.65 (1−6; SD 1.17), with nearly half (*n*=12; 48%) seeing at least two different clinicians to complete their treatment (range 1−5; SD 1.29). Nineteen cases (76%; CI 59–82%) met the ‘satisfactory’ technical quality criteria (Fig. 1b). Of the six (24%) unsatisfactory cases, the apical plug was either >1mm from the radiographic apex (*n*=3; 12%) or overextended (*n*=3; 12%) with four cases (16%) showing non-homogenous density with voids present at the apex. Over a third of cases (*n*=9; 36%) had apical plugs >4mm in length, with ten cases (40%) where the obturation material extended above the level of the CEJ. Overall success rate at 12-month follow-up was 88%, with only three patients (12%) demonstrating clinical signs and symptoms of failure including presence of swelling, sinus and mobility. None of the failed treatments were re-treatments. Coronal discolouration was present in three cases (12%).

### TotalFill Putty

Of the 25 cases analysed, mean patient age was 10.6 years (8−11; SD 1.91), with a relatively even split of male (*n*=13; 52%) and female (*n*=12; 48%) patients. While the mean time from new patient appointment to extirpation was 24 days (0−28; SD 5.29), the overall mean treatment period was 87 days (SD 37.2). Maxillary central incisors were the most treated teeth (n=19; 76%) with the most common trauma sustained complicated crown fractures (*n*=11; 44%). All cases were primary endodontic treatments (*n*=25; 100%). The mean number of appointments was 2.8 (1−4; SD 1.33), with patients seeing an average of two different clinicians for completion of treatment (1−4; SD 1.48). Twenty-four (96%) cases were of ‘satisfactory’ technical quality (Fig. 1c)—in the unsatisfactory case (*n*=1, 4%), the plug was: >1mm from the radiographic apex; and overextended, with a non-homogenous density and voids elsewhere in the obturation. All cases had at least 4mm of apical plug length, with seven cases (28%) having excess plug length (>5mm) and six cases (24%) with obturation material (gutta-percha [GP]) above the level of the CEJ. Overall success rate at 12-month follow-up was 92%, with two cases (8%) demonstrating signs and symptoms of failure—sinus, swelling, and tenderness to percussion/palpation, as well as an increasing PARL.

### Patient cooperation

Relative analgesia (RA), in the form of inhalation sedation (IHS), was used for those children who exhibited signs of dental anxiety and/or cooperation challenges, to allow successful administration of local anaesthesia (LA) during the first phase of endodontic treatment, i.e., pulp extirpation (*n*=9; 12%). Reduced cooperation, i.e., those who exhibited challenging behaviour, such as repetitive movement during treatment, was statistically significant and led to unsatisfactory technical quality, in comparison to those who managed well (X^2^ [1, *n*=7] = 3.183, *p*=0.021). RA was not used for the placement of any apical plugs and its initial use produced no statistically significant benefit in terms of the clinical outcome of treatment (X^2^ [1, *n*=9] = 3.183, *p*=0.642).

### Local anaesthesia and obturation

There was a statistically significant association between local anaesthesia use and apical plug placement, with non-use of local anaesthetic during the obturation appointment associated with an apical plug being >1mm from the apex (X^2^ [1, *n*=13] = 6.319, *P* = 0.028). There was no statistically significant association found between local anaesthesia use and apical plug placement, with non-use of local anaesthetic during the obturation appointment not associated with an apical plug being >2mm from the apex (X^2^ [1, *n*=61] = 2.239, *P* = 0.287). Overextension of the apical plug material past the radiographic apex was not significantly associated with treatment failure, i.e., no negative effect on healing or reduction of PARL (*p*=0.347).

### Regression analysis

A logistic regression was conducted to assess whether the risk model, with seven predictor variables, was able to determine if there was a significant relationship to treatment failure (the outcome variable). The model considered the treatment failures collectively, rather than independently per bioceramic material and was statistically significant when all predictor variables were considered together: χ^2^=93.27 (6); *p* value<0.001. The model explained 79.5% (Nagelkerke *R*^*2*^) of the variance of treatment failures and correctly classified 96% of cases. Involvement of more than two operators in treatment was statistically significant and nearly two-and-a-half times more likely to result in treatment failure (Table 5). An apical plug of less than 4mm thickness was around 1.5 times more likely to result in failure; however, this was not statistically significant. The model revealed a statistically significant relationship between a non-consultant (i.e., non-senior) grade performing the apical plug and obturation (*p*=0.004; OR 2.141) and treatment being completed in more than two visits (*p*=0.006; OR 2.542). The presence of pre-operative root resorption was associated with the highest likelihood of treatment failure (*p*=0.003; OR 3.522).

**Table 5:** Multivariate logistic regression model of factors associated with endodontic treatment failure

Variable	Regression coefficient (β)	OR	95% CI		*P* value
			Lower	Upper	
*Number of Operators*					
>2 ≤2	0.632 *Reference*	2.483 1	1.198 –	3.547 –	0.005* –
*Clinician – Apical plug and Obturation Visit*					
Non-Consultant Consultant	0.877 *Reference*	2.141 1	1.217 –	3.732 –	0.004* –
*Final Restoration*					
Crown former Palatal composite	0.579 *Reference*	1.815 1	0.635 –	2.891 –	0.093 –
*Number of treatment appointments*					
>2 2	0.733 *Reference*	2.542 1	1.081 –	2.938 –	0.006* –
*Apical Plug Thickness*					
<4mm ≥4mm	0.511 *Reference*	1.549 1	0.760 –	2.416 –	0.018 –
*Apical plug distance from radiographic apex*					
>1mm ≤1mm	0.411 *Reference*	1.682 1	0.974 –	2.724 –	0.072 –
*Pre-operative resorption*					
Yes No	0.645 *Reference*	3.522 1	1.327 –	4.196 –	0.003* –

Hosmer and Lemeshow Test: χ^2^=93.27; df=6; *p-*value<0.001

*OR* Odds Ratio, *CI* Confidence Interval, *df* degrees of freedom

*p value <0.007 following Bonferroni correction denotes statistical significance

## Discussion

To the best of the authors’ knowledge, this is the first study to evaluate the technical quality and clinical outcome of apexification procedures in children of three separate bioceramic materials. Our clinical outcome results indicate that MTA (84%), Biodentine (88%), and TotalFill Putty (92%) are extremely effective apexification materials for immature permanent incisors following 12-month review—radiographic changes (healing) are not typically evident until at least 6 months post-obturation, and hence, the ESE (2006), recommend that root canal treatment should be assessed at least 1 year post-treatment, then as required for up to 4 years thereafter. Our results are comparable (and superior for Biodentine and TotalFill) to those in the adult literature (Clarke et al. 2015, Farzaneh et al. 2004, Friedman et al. 2003, Kaur et al. 2017, Moore et al. 2011, Ng et al. 2011a, Ng et al. 2011b). In addition, the technical quality of each was ’satisfactory’ in 80% of MTA, 76% of Biodentine, and 96% of TotalFill Putty cases at 12-month review.

The significant reduction in treatment time for bioceramics has reduced the risk of microleakage and reinfection of the canal due to no longer placing multiple interim coronal restorations. A one-visit approach for plug placement and obturation (following an initial extirpation visit) also limits the amount of time taken off work and school for children and their guardians (Albadri et al. 2013, Dania and Prisinda, 2022). Previous protocols insinuate a significant treatment burden as between 2 and 25 visits were necessary to achieve barrier formation with calcium hydroxide prior to obturation (Kinirons et al. 2001)—travel to specialist centres for apical plug placement with bioceramics may actually be more cost-effective, despite the higher cost of the individual material (Table 1) and longer distance to travel—a detailed cost–benefit analysis, however, would be required to draw any firm conclusions (Nayar et al. 2009).

The use of calcium hydroxide is not entirely defunct, however, as due to the presence of thin, fracture-prone dentinal walls, disinfection of the root canal system still relies heavily on chemical disinfection using irrigants and intracanal medicaments, of which calcium hydroxide is still the most popular (Parolia et al. 2022). It has been shown to be effective against the most common endodontic pathogens (Mohammadi and Dummer 2011, Oliveira et al. 2008), and a recent systematic review by Wikström et al. (2021) highlighted that, on average, canals are typically ‘dressed’ with calcium hydroxide between 1 and 6 weeks following extirpation, prior to apical plug placement—our mean duration of calcium hydroxide dressing for results for MTA (7 weeks) and Biodentine (7 weeks) is similar to those by Moore et al. (2011) (8.5 weeks), with the average time of calcium hydroxide dressing for TotalFill Putty (9 weeks) slightly longer due to initial limitations on aerosol generating procedures as a result of the COVID-19 pandemic.

Sodium hypochlorite (NaOCl) is an excellent irrigant which dissolves organic pulp tissue and possesses broad-spectrum antimicrobial activity against endodontic microorganisms and biofilms (Lee et al. 2015)—a systematic review by Arruda and colleagues (2019) found that various concentrations (0.5–5.25%) have excellent non-specific proteolytic activity, with insufficient research on the optimal time, volume, or concentration for endodontic treatment, without causing significant changes to the dentinal mechanical properties (Parolia et al. 2022). Our department protocol (Box 1) utilises 5.25% NaOCl, as it has been shown to have a greater antimicrobial activity, an improved ability to dissolve necrotic tissue, and a reduced effectiveness time, compared to 2.5%, i.e., more efficient action over a shorter period of time, something incredibly important when treating children (Marion et al. 2012, Whyatt et al. 2024).

All endodontic treatment must be performed under rubber dam isolation. Isolation is commonly achieved using a non-latex rubber dam, such as DryDam (Directa, Upplands Väsby, Sweden), interproximal Wedjets (Coltène/Whaledent Ltd, West Sussex, UK), and a gingival barrier such as Oraseal (Ultradent, South Jordan, Utah, USA) or liquid dam (Cerkamed, Poland) (Baughan et al. 2019)—this negates the need for conventional endodontic clamp isolation and LA, which many children find the most anxiety-provoking part of treatment (Chandraweera et al. 2019). A patient’s ears hold the DryDam in place, much like an ear loop face mask, and it is very well accepted by paediatric patients (McKay et al. 2013, Vanhée et al. 2021), especially given the normalisation of mask wearing following the COVID-19 pandemic.

To ensure accurate instrumentation, disinfection, apexification, and obturation of the root canal, establishment of the working length (WL) is critical. While the British Endodontic Society (BES) advise this is best determined using an electronic apex locator (EAL) (Tomson 2022), this is challenging in immature teeth as not only has the lack of an apical constriction been shown to produce unreliable readings, but canals wider than ISO 80 have demonstrated poor reliability, with the thought process being that excessive apical tissue fluid present in open apices may affect the accuracy of the readings produced (Clarke et al. 2015, Kim and Chandler 2013). As sensibility testing has been shown to provide unreliable results in children, especially in immature teeth, local anaesthesia is normally utilised for pulp extirpation where tests have shown conflicting results (Berman and Hargreaves 2020, Jafarzadeh and Abbott 2010, Nagarathna et al. 2015). Although this may create additional management challenges for treatment, it counters the known fear and apprehension associated with canal instrumentation in anxious children—LA was used in 88% (*n*=72) of pulp extirpations in this evaluation. Only 11 patients (14.7%) had LA administered during their apexification visit, however, and our results found no statistically significant association between local anaesthesia use and apical plug placement, i.e., non-use of local anaesthetic during the obturation appointment was not associated with an apical plug being >2mm from the radiographic apex (*p*=0.287) and thus still within accepted limits (Hadjiantonis 2016).

The pulp canal space of a traumatised non-vital tooth requires immediate disinfection, as the susceptibility to root resorption in immature teeth makes this process occur rapidly due to the wide dentinal tubules which allow penetration of bacteria (O'Reilly and Tanday 2019, Oliveira et al. 2008). Inadequately cleaned canals, especially those associated with prolonged periods of time between extirpation and obturation appointments, have the potential to undergo inflammatory and/or external root resorption, which may lead to altered apical anatomy (and thus an altered working length)—our results indicate that the presence of pre-operative root resorption was associated with 3.5 times increased odds of treatment failure (*p*=0.003). As such, the authors recommend re-confirmation of the working length prior to apexification, especially if: there has been an extended period of time between appointments; a different operator is performing the apexification; the original working length needed adjustment; navigation of the canal produces a different working length; the patient reports feeling the file at a point shorter than the previous working length; or if the coronal restoration has been lost or replaced between appointments thus changing the previously known length and reference point. This may or may not be performed under LA.

The length of the apical plug has also been associated with satisfactory technical quality and clinical outcomes. A minimal plug length of 4–5 mm is now advised for all bioceramics when used for apexification (Abbas et al. 2020, Clarke et al. 2015, Dania and Prisinda, 2022, Kumar et al. 2014, Mente et al. 2013). The evidence implies this offers the optimal sealing ability, least risk of microleakage, and significantly more displacement resistance than 3mm plugs, with the recent studies finding TotalFill Putty exhibited significantly higher bond strength to dentine than MTA and Biodentine (Kadić et al. 2018, Raju et al. 2022). In this study, MTA was the most common material to have less than 4mm of apical plug length, with less-experienced clinicians more likely to not only have an apical plug short of the desired distance, but also short of the desired length, possibly due to the difficult handling and manipulation of MTA reported in the literature (Ayub and Darcey 2023)—our results indicate that a plug length of less than 4mm was 1.5 times more likely to result in treatment failure.

The choice of restoration, which simultaneously provides the coronal seal, depends on the extent of tooth tissue lost as a result of the original trauma. Cellulose crown formers (also termed ‘strip crowns’) are routinely used to restore traumatised anterior teeth which have suffered multi-surface tissue loss (O'Reilly and Tanday 2019), as they maintain an effective coronal seal, restore interim function, and reinstate aesthetics (Sarao et al. 2021). Despite reinstating a degree of function anteriorly, patients should be cautioned against using heavily restored root-filled anterior teeth for heavy loading and eating hard foods, as large anterior composite restorations are subjected to significant amounts of stress during occlusal function, and therefore have been shown to have a poorer long-term prognosis than other restorations and failure of treatment due to loss of the coronal seal—our results agree with those in the literature, as restoration with a crown former for multi-surface tissue loss was associated with a 1.8 times higher odds of treatment failure when compared with those without extensive tooth tissue loss and only a palatal composite in the access cavity (Liddelow and Carmichael 2016, Spinas 2004).

Clinician experience appears to be a key prognostic factor in relation to successful clinical outcomes and also for technical quality. Our results indicate that treatment failure is around twice as likely (*p*=0.004; OR 2.141) when a non-consultant (i.e., non-senior) grade places the apical plug and obturation. These findings concur with the results of other studies where more junior clinicians or those with limited operator experience and skill, have poorer clinical outcomes, especially where MTA has been used (Di Filippo et al. 2014, Mente et al. 2013, Ng et al. 2011a). In addition, where a patient had extirpation performed by a junior clinician, and required more than two appointments to complete treatment (e.g., if an interim restoration was lost between visits and the tooth subsequently required repeat disinfection and replacement of the restoration), they were at an increased likelihood for treatment failure (*p*=0.006; OR 2.542). Patients presenting with issues such as lost restorations tend to present acutely and may not be managed by the same clinician who performed the first (extirpation) or final treatment (apexification and obturation), highlighting the importance of continuity of care and experience, as multiple visits, treatment by junior clinicians, and treatment by multiple different clinicians had a significant association on treatment failure and poorer technical outcomes.

## Strengths and limitations

The main limitation of the paper lies in the study design itself. The data collected represent a cross-sectional analysis of a service evaluation of the ‘real-life’ clinical outcomes of a specialist paediatric department providing endodontic treatment, and not that of a randomised-controlled trial with predetermined, fixed protocols. Hence, the findings may not be wholly generalisable to wider dental services. There may also be a risk of case selection bias, as for the sake of time and brevity, cases were not chosen at random; however, efforts were made to negate such a bias by chronologically choosing cases to reflect the modern treatment methods employed in the department. No recognised scale or index was used for radiographic assessment; however, the thorough methodology employed showed almost perfect intra- and inter-examiner agreement—future studies should ensure that a recognised measure such as the complex periapical index (COPI), periapical index (PAI), or the endodontically treated tooth index (ETTI) is utilised, as this will allow more thorough comparison between other studies in the literature (Ballikaya et al. 2022). Although the follow-up period was only for 12 months, this was in line with national and international guidance and showed excellent technical quality and clinical outcomes for all materials evaluated. In addition, given the volume of trauma which the department sees, follow-up periods >12 months would not be feasible for every patient. Furthermore, this study has numerous additional strengths and is the first to provide an estimated cost-per-tooth for each material, based on: gross manufacturer costs per material capsule/syringe, average apical size/volume, and the provision of a 4mm apical plug (Table 1). Cost knowledge can also have economic benefits for healthcare providers and provide a more cost-effective option for use in general dental practice or salaried dental services. No other papers comparing the technical quality and clinical outcomes of different bioceramic apical plug materials in the paediatric population exist in the evidence base; hence, despite not being an RCT, this study serves as a very useful baseline for future research and comparison to other specialist centres across the UK and beyond.

## Conclusions

The use of MTA, Biodentine, and TotalFill Putty produced excellent clinical outcomes for apexification that are of both patient and clinician interest. Given the high success rates of all materials, personal preference, training, and experience may subjectively play a part in material choice. A number of variables and trends highlight how logistical and situational factors, such as continuity of care from operators with increased skill and experience, may play a key role in technical quality and clinical outcomes, rather than individual material choice, as treatment with junior clinicians, alongside treatment by different clinicians across a course of treatment, had a significant association with treatment failure and poorer technical outcomes.

## Supplementary Information

Below is the link to the electronic supplementary material.

Supplementary file1 (DOCX 21 KB)

## Data Availability

Due to ethical concerns, supporting data cannot be made openly available. Further information about the data and conditions for access are available from the corresponding author upon reasonable request.
