# Supplementary material for: Comparing the technical quality and clinical outcomes of root canal treatment on immature permanent incisors in children: a retrospective evaluation of three bioceramic plug materials
Source: Eur Arch Paediatr Dent. 2024 Sep 10;25(6):821–35. doi: 10.1007/s40368-024-00941-3 (PMC11576660; doi:10.1007/s40368-024-00941-3)
Supplement: Supplementary file 1 — Supplementary file1 (DOCX 21 KB) [file 40368_2024_941_MOESM1_ESM.docx]

***Supplementary Table 1. Clinical outcomes for each treatment group.***

| **Variable** | **Category** | **MTA**  **(N/%)** | **Biodentine**  **(N/%)** | **TotalFill Putty**  **(N/%)** |
| --- | --- | --- | --- | --- |
| **CLINICAL/RADIOGRAPHIC ASSESSMENT** | | | | |
| **Pre-Treatment** | | | | |
| **Resorption** | **Yes** | 2 (8) | 3 (12) | 3 (12) |
|  | **No** | 5 (20) | 4 (16) | 2 (8) |
|  | **Not Recorded** | 18 (72) | 18 (72) | 20 (80) |
| **PARL** | **Yes** | 5 (20) | 8 (32) | 10 (40) |
|  | **No** | 5 (20) | 4 (16) | 9 (36) |
|  | **Not Recorded** | 15 (60) | 13 (52) | 6 (24) |
| **12-month Follow-Up** | | | | |
| **Symptoms** | **Yes** | 4 (16) | 3 (12) | 2 (8) |
|  | **No** | 21 (84) | 21 (84) | 23 (92) |
|  | **Not Recorded** | 0 (0) | 1 (4) | 0 (0) |
| **Clinical Signs** | **TTP** | 4 (16) | 3 (12) | 2 (8) |
|  | **Swelling** | 3 (12) | 1 (4) | 1 (4) |
|  | **Sinus** | 4 (16) | 2 (8) | 1 (4) |
|  | **Discolouration** | 7 (28) | 3 (12) | 3 (12) |
|  | **Mobility** | 0 (0) | 2 (8) | 1 (4) |
| **Resorption** | **Yes** | 3 (12) | 4 (16) | 3 (12) |
|  | **No** | 20 (80) | 19 (76) | 20 (80) |
|  | **Not Recorded** | 2 (8) | 2 (8) | 2 (8) |
| **Size of PARL** | **Unchanged** | 1 (4) | 1 (4) | 1 (4) |
|  | **Increasing** | 4 (16) | 3 (12) | 2 (8) |
|  | **Decreasing** | 5 (20) | 5 (20) | 4 (16) |
|  | **Not Present** | 15 (60) | 16 (64) | 18 (72) |
| **Outcome** | | | | |
| **Discharge Outcome** | **Healed** | 15 (60) | 16 (64) | 18 (72) |
|  | **Healing** | 5 (20) | 5 (20) | 4 (16) |
|  | **Continuing Disease (Survival)** | 1 (4) | 1 (4) | 1 (4) |
|  | **Failure** | 4 (16) | 3 (12) | 2 (8) |
| **Discharge Advice** | **No further treatment required** | 12 (48) | 15 (60) | 15 (60) |
|  | **Monitor ongoing healing** | 6 (24) | 6 (24) | 4 (16) |
|  | **Place definitive restoration** | 0 (0) | 0 (0) | 1 (4) |
|  | **Other** | 7 (28) – Other restorative treatment | 4 (16) – Other restorative treatment | 5 (20) – Other restorative treatment |
| **Key**:  PARL – periapical radiolucency \| TTP – tenderness to percussion/palpation | | | | |
